# Unravelling the complex trait of harvest index in rapeseed (*Brassica napus* L.) with association mapping

**DOI:** 10.1186/s12864-015-1607-0

**Published:** 2015-05-12

**Authors:** Xiang Luo, Chaozhi Ma, Yao Yue, Kaining Hu, Yaya Li, Zhiqiang Duan, Ming Wu, Jinxing Tu, Jinxiong Shen, Bin Yi, Tingdong Fu

**Affiliations:** National Key Laboratory of Crop Genetic Improvement, National Center of Rapeseed Improvement in Wuhan, Huazhong Agricultural University, Wuhan, 430070 P.R. China

**Keywords:** Harvest index, Complex traits, *Brassica napus*, Association mapping, Correlation

## Abstract

**Background:**

Harvest index (HI), the ratio of grain yield to total biomass, is considered as a measure of biological success in partitioning assimilated photosynthate to the harvestable product. While crop production can be dramatically improved by increasing HI, the underlying molecular genetic mechanism of HI in rapeseed remains to be shown.

**Results:**

In this study, we examined the genetic architecture of HI using 35,791 high-throughput single nucleotide polymorphisms (SNPs) genotyped by the Illumina BrassicaSNP60 Bead Chip in an association panel with 155 accessions. Five traits including plant height (PH), branch number (BN), biomass yield per plant (BY), harvest index (HI) and seed yield per plant (SY), were phenotyped in four environments. HI was found to be strongly positively correlated with SY, but negatively or not strongly correlated with PH. Model comparisons revealed that the A–D test (ADGWAS model) could perfectly balance false positives and statistical power for HI and associated traits. A total of nine SNPs on the C genome were identified to be significantly associated with HI, and five of them were identified to be simultaneously associated with HI and SY. These nine SNPs explained 3.42 % of the phenotypic variance in HI.

**Conclusions:**

Our results showed that HI is a complex polygenic phenomenon that is strongly influenced by both environmental and genotype factors. The implications of these results are that HI can be increased by decreasing PH or reducing inefficient transport from pods to seeds in rapeseed. The results from this association mapping study can contribute to a better understanding of natural variations of HI, and facilitate marker-based breeding for HI.

**Electronic supplementary material:**

The online version of this article (doi:10.1186/s12864-015-1607-0) contains supplementary material, which is available to authorized users.

## Background

Yield in rapeseed (*Brassica napus* L.; AACC, 2n = 38) has attracted the interest of plant breeders for many years. In the past decades, the productivity levels have increased due to the extensive use of heterosis in hybrid breeding. However, the average heterosis percentage for vegetative biomass, seeds per pod and 1,000-seed weight are only 25–30 %, 17.5 %, and 1.8 %, respectively [1]. Leaves, pods, and other above-ground green tissues are able to photosynthesize as “source” organs, while seeds are storage organs that serve as the “sink” for photosynthetic products. The usual incomplete development filling of seeds and the low heterosis rate of seed weight suggest that when the “sources” are in surplus, the sinks are not fully filled or utilized. This could be an indication of unsmooth limitation in the “flow”, resulting in lower heterosis in seed weight. Chhabra [2] and Shen *et al.* [3] observed that when the source and sink organs are not limiting, and the translocation of assimilates is the most critical limiting factor for seed yield in *Brassica*. Similar observations were reported for rice and triticale [4]. Therefore, the balance among photosynthetic “source”, “flow” and “sink” is critical for yield improvement; this balance can be evaluated using harvest index (HI) as the criterion.

The HI is the ratio of grain yield to total biomass (usually the total above–ground biomass). This index is considered as a measure of the biological success in partitioning assimilated photosynthate to the harvestable product [5, 6]. As an integrative trait, HI was identified to be highly correlated with a number of yield-related traits in important crop species such as rice [7, 8] and sorghum [9–11]. Generally, the correlated traits are interrelated, and an increase in one component may lead to a decrease or an increase in others. Interestingly, the increase in HI almost fully accounted for the progressive increase in the grain yield potential of wheat, barley and rice between 1900 and 1980 [12]. Thus, a better understanding of the genetic mechanism of HI is crucial for interpreting agronomically important characters such as yield.

Association mapping, also called linkage disequilibrium (LD) mapping, utilizes the large number of historical recombination events that have occurred throughout the entire evolutionary history of the mapping population, allowing fine-scale QTL mapping [13, 14]. Very recently, association mapping performed with genomic, transcriptomic, epigenetic and metabolic data has provided abundant information on the genetic architecture of complex quantitative traits in a number of crop species such as maize [15–19], *Arabidopsis* [20], rapeseed [21–23], and rice [24, 25]. However, the false positive rate caused by population structure is difficult to predict. Several methods have been proposed to deal with this problem. Aranzana *et al.* [26] found that removing the genetically distinct and phenotypically extreme accessions for flowering time could significantly reduce the false positive rate in *Arabidopsis*. Huang *et al.* [24] successfully corrected the elevated false positive rate by developing an analytical framework for haplotype-based *de novo* assembly of low-coverage sequencing data and identified candidate genes for 18 associated loci through detailed annotation in rice. Li *et al.* [21] and Cai *et al.* [22] controlled false associations in association mapping for seed quality and yield-related traits in rapeseed by using model comparisons (GLM, Q, PCA, K, PCA+K and Q+K model). Yang *et al.* [27] studied the genetic architecture of 17 agronomic traits in an enlarged maize association panel by a new nonparametric model, the Anderson–Darling (A–D) test, also known as the ADGWAS model, and found that the false positives and statistical power were efficiently balanced. Additionally, joint linkage-association mapping strategies were proposed to evaluate the false association in soybean [28]. These reports suggested that association mapping will be a powerful approach for exploring the QTLs responsible for HI.

In the present study, a genome-wide association study (GWAS) of five traits (plant height (PH), seed yield per plant (SY), biomass yield per plant (BY), branch number (BN) and HI) was performed with a panel of 155 accessions using 35,791 genomic SNPs from the Illumina BrassicaSNP60 Bead Chip. To control spurious associations, we analyzed the genetic population structure and familial relatedness in the GWAS population. Seven different mapping models were tested for the best fit of each trait. The chosen model was used to map markers associated with the five traits phenotyped in four environments. The objectives of the present study were to: (1) obtain a better understanding of HI of inbred lines; (2) examine the relationship between HI and other traits; (3) perform association mapping for the five traits; and (4) discuss the implications of the results of this study for further marker-assisted selection breeding in *B. napus*.

## Methods

### Plant materials and field experiments

The genetic population consisted of 155 genetically diverse inbred lines (Additional file 1: Table S1). All the accessions were provided by the National Research Center of Rapeseed Engineering and Technology, Huazhong Agricultural University, Wuhan, China.

The 155 inbred lines were grown in a randomized complete block design with two replications in four different environments: Huanggang (32.27° N, 114.52° E) and Xiangyang (32.01° N, 112.08° E) in the 2011/2012 growing season; and Wuhan (29.58° N, 113.53° E) and Xiangyang (32.01° N, 112.08° E) in the 2012/2013 growing season. All four sites were located along the middle reaches of the Yangtze River in China. For convenience, the four sites are hereafter referred to as E1, E2, E3 and E4. A plot size of three rows (12 plants per row) was used with two replications. A spacing of 30 × 20 cm between rows and between plants within a row was used at all locations. The management of the field experiments was performed in accordance with local standard practices. In each plot, PH, BN, BY and SY were measured for five representative *B. napus* plants at maturity. The HI (%) was calculated as the ratio of SY to BY.

### Genotype and data analyses

The whole population of inbred lines was genotyped using the Brassica 60 K Illumina® Infinium SNP array by Emei Tongde Co. (Beijing) according to the manufacturer’s protocol (http://www.illumina.com/technology/beadarray-technology/infinium-hd-assay.html). The SNP data were clustered and called automatically using Illumina BeadStudio genotyping software. Those SNPs with either AA or BB frequency equal to zero (*i.e.*, monomorphic), call frequency < 0.9, or minor frequency < 0.05 were excluded.

The data for the five traits were tested by analysis of variance (ANOVA) using *SPSS* version 19.0 (IBM Corp., Armonk, NY, USA).

### Genetic diversity and population structure analysis

The population structure was inferred using the software package *STRUCTURE* v2.3.4 [29]. Five independent runs were performed with a *K*-value (the putative number of genetic groups) from 1 to 10, with the length of burning period and the number of MCMC (Markov Chain Monte Carlo) replications after burning both set to 100,000 iterations under the ‘admixture model’. The most likely *k*-value was determined by the log probability of data [LnP(D)] and an ad hoc statistic Δk based on the rate of change of LnP(D) between successive k values as described by Evanno *et al.* [30]. The cluster membership coefficient matrices of replicate runs from *STRUCTURE* were integrated to obtain the Q matrix using *CLUMPP* software [31] and graphically displayed using *DISTRUCT* software [32]. Nei’s genetic distance [33] was estimated and used to construct an unrooted neighbor-joining tree representing the genome-wide relationship among the accessions. The tree was constructed using the Unweighted Pair-Group Method with Arithmetic mean (UPGMA) method with *PowerMarker* software. The tree was visualized using *FigTree* (http://tree.bio.ed.ac.uk/software/figtree/). The genetic relatedness between individuals was estimated by principal component analysis (PCA) using *NTSYSpc* version 2.11 [34].

### Genome-wide association analysis

The effects of population structure (Q, PC) and kinship (K) on the HI-related traits were evaluated by GWAS using seven models: (i) GLM, without controlling for Q and K; (ii) Q model, controlling for Q; (iii) PCA model, controlling for PC, with the top two principal components used as fixed effects; (iv) K model, controlling for K; (v) PCA+K model, controlling for both PC and K; (vi) Q+K model, controlling for both Q and K; and (vii) ADGWAS model, controlling for Q. The GLM, Q and PCA models were performed using a general linear model (GLM); the K, PCA+K and Q+K models were performed using a mixed linear model (MLM) with optimum compression and population parameters previously determined (P3D) by variance component estimation in TASSEL 3.0 [35, 36]. The A–D [27] test, also known as the nonparametric model or ADGWAS model, was performed using an *R* script, ADGWAS (http://www.maizego.org/Resources.html). Statistically significant loci were identified by comparing P values with the Bonferroni threshold (1/35791 = 2.79E-5).

### Phenotypic variation explained by multiple SNPs

Stepwise regression was performed to examine the effect of multiple alleles with different functional polymorphisms on the HI traits, and to estimate the total variance explained (*R*^*2*^), using the *lm* function in *R* [37].

## Results

### Phenotypic variations for the five traits

Table 1 lists the details of the phenotypic variations of the five traits (PH, BN, SY, BY and HI). The five traits were normally distributed in the population (except for BY in E2). In the four environments, HI ranged from 0.15 to 0.36 with an average of 0.20 to 0.27. Comparatively, HI in E4 showed the lowest coefficient of variation (9.71 %), while SY in E3 had highest coefficient of variation (34.90 %) among all five traits.

**Table 1:** Phenotypic variations of the five traits

Trait	Env.	Mean Ậ ± SD	Range	Skew	Kurt	CV%
PH	E1	*	*	*	*	*
	E2	*	*	*	*	*
	E3	125.87 ± 0.788	97.00–156.70	−0.03	0.33	11.17
	E4	128.77 ± 0.956	93.71–155.89	−0.48	0.39	12.41
BN	E1	6.91 ± 0.081	4.40–9.25	0.07	−0.31	16.78
	E2	6.15 ± 0.072	4.49–8.90	0.70	0.27	16.61
	E3	4.42 ± 0.083	4.20–7.80	0.10	0.48	24.28
	E4	7.56 ± 0.106	4.46–12.05	0.31	0.56	19.44
BY	E1	43.53 ± 0.943	21.63–71.74	0.38	−0.38	26.45
	E2	29.79 ± 0.642	15.86–77.86	1.77	8.38	26.35
	E3	46.54 ± 0.725	22.00–71.50	0.34	−0.07	19.01
	E4	41.03 ± 0.888	16.34–78.04	0.48	0.53	26.41
SY	E1	9.68 ± 0.265	4.11–18.11	0.34	−0.54	33.39
	E2	8.09 ± 0.179	4.12–19.47	1.08	1.82	26.95
	E3	9.67 ± 0.251	4.85–20.79	1.03	1.31	34.90
	E4	9.84 ± 0.236	4.38–18.16	0.44	−0.15	29.24
HI	E1	0.22 ± 0.003	0.15–0.33	0.43	−0.29	17.18
	E2	0.27 ± 0.003	0.17–0.34	−0.55	0.33	11.54
	E3	0.20 ± 0.004	0.15–0.36	1.32	2.94	20.06
	E4	0.24 ± 0.002	0.19–0.30	0.06	−0.38	9.71

Trait: *PH* plant height, *BN* branch number, *BY* biomass yield per plant, *SY* seed yield per plant, *HI* harvest index.

Env: *E1* Huanggang in 2011, *E2* Xiangyang in 2011, *E3* Wuhan in 2012, *E4* Xiangyang in 2012.

*SD:* standard deviation. *CV(%):* coefficient of variation.

*: data not collected.

Two-factor ANOVA suggested that the differences caused by genotypes and environments were significant at the 0.05 and 0.01 levels, respectively, for all of the complex traits (Additional file 2: Table S2). There was not a strong correlation, or a negative correlation, between HI and PH (−0.29 at p = 0.01 level at E3, 0.01 at E4), but a strong positive (P = 0.01) correlation between HI and SY (0.34–0.83 across the four different environments). The BN and BY were significantly positively (P = 0.01) correlated with HI in E3, but the correlations were relatively weak in the other environments (Additional file 3: Table S3).

### Genetic diversity and population structure analysis

**Fig. 1:**
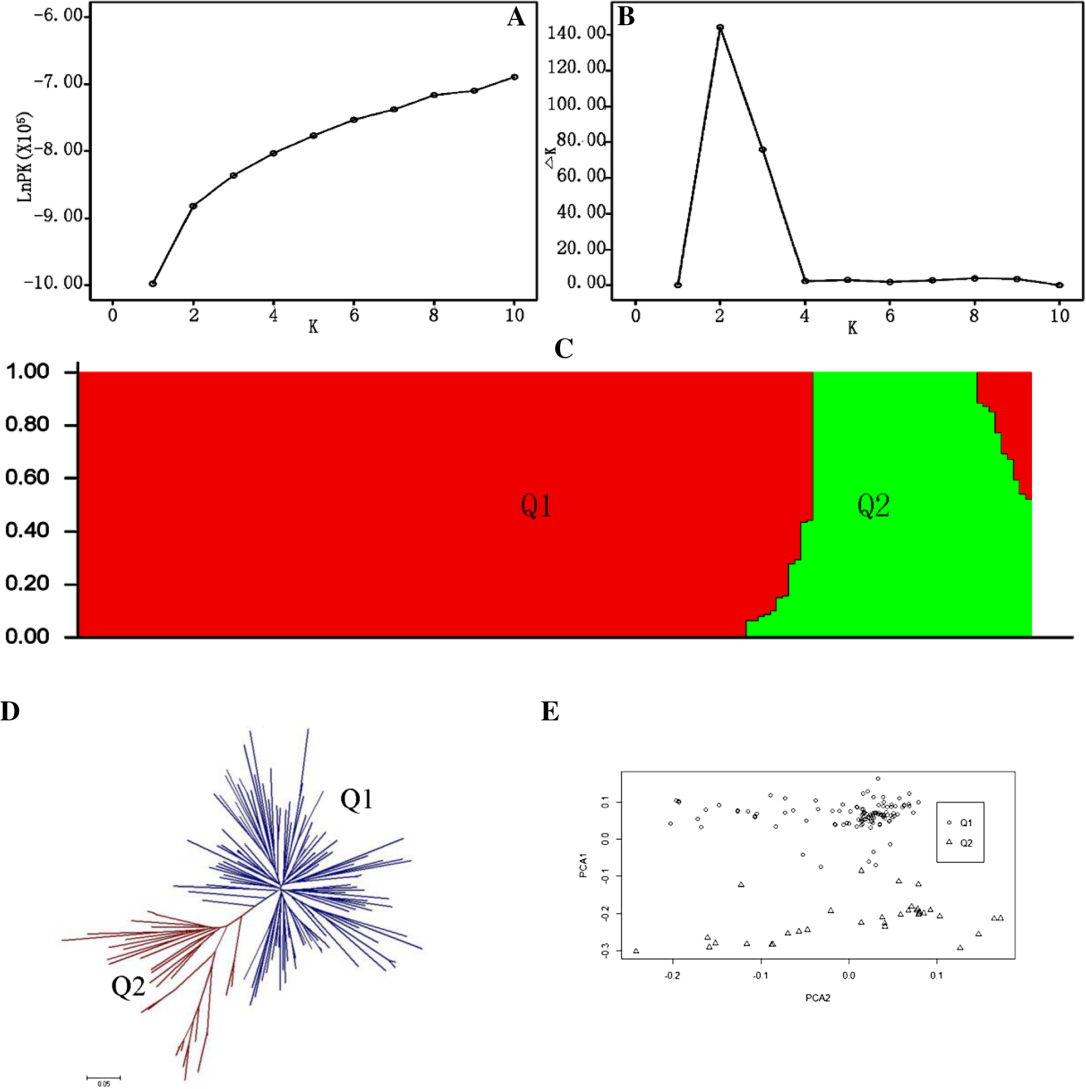
Analysis of population structure of 155 rapeseed accessions using STRUCTURE and Unrooted UPMGA. (**A**) Estimated LnP(K) of possible clusters (k) from 1 to 10. (**B**) ΔK based on rate of change of LnP (K) between successive K values. (**C**) Population structure based on *k* = 2. Red represents Subgroup Q1; green, Subgroup Q2. (**D**) Dark blue, Subgroup Q1; fuchsia, Subgroup Q2. (**E**) Principal components analysis (PCA)

The population structure of the 155 accessions was identified based on 7,600 SNPs using *STRUCTURE* software (Fig. 1). Clustering inference performed with possible clusters (K) from 1 to 10 showed that the most significant change of likelihood occurred when K increased from 2 to 3, and the highest Δk value was observed at k = 2 (Fig. 1A, B, C). A radial tree created with *PowerMarker* had two main branches for the 155 accessions (Fig. 1D). The PCA also displayed the pattern of the genetic structure of the GWAS population (Fig. 1E). All the parameters suggested that the two-group model (subgroups Q1 and Q2) sufficiently explained the genetic structure among the 155 accessions, and inspection also confirmed that the phenotypes were not randomly distributed with respect to this genetic structure. Altogether, 118 accessions belonged to subgroup Q1, and 37 accessions belonged to subgroup Q2.

### Model comparisons for controlling false associations

**Fig. 2:**
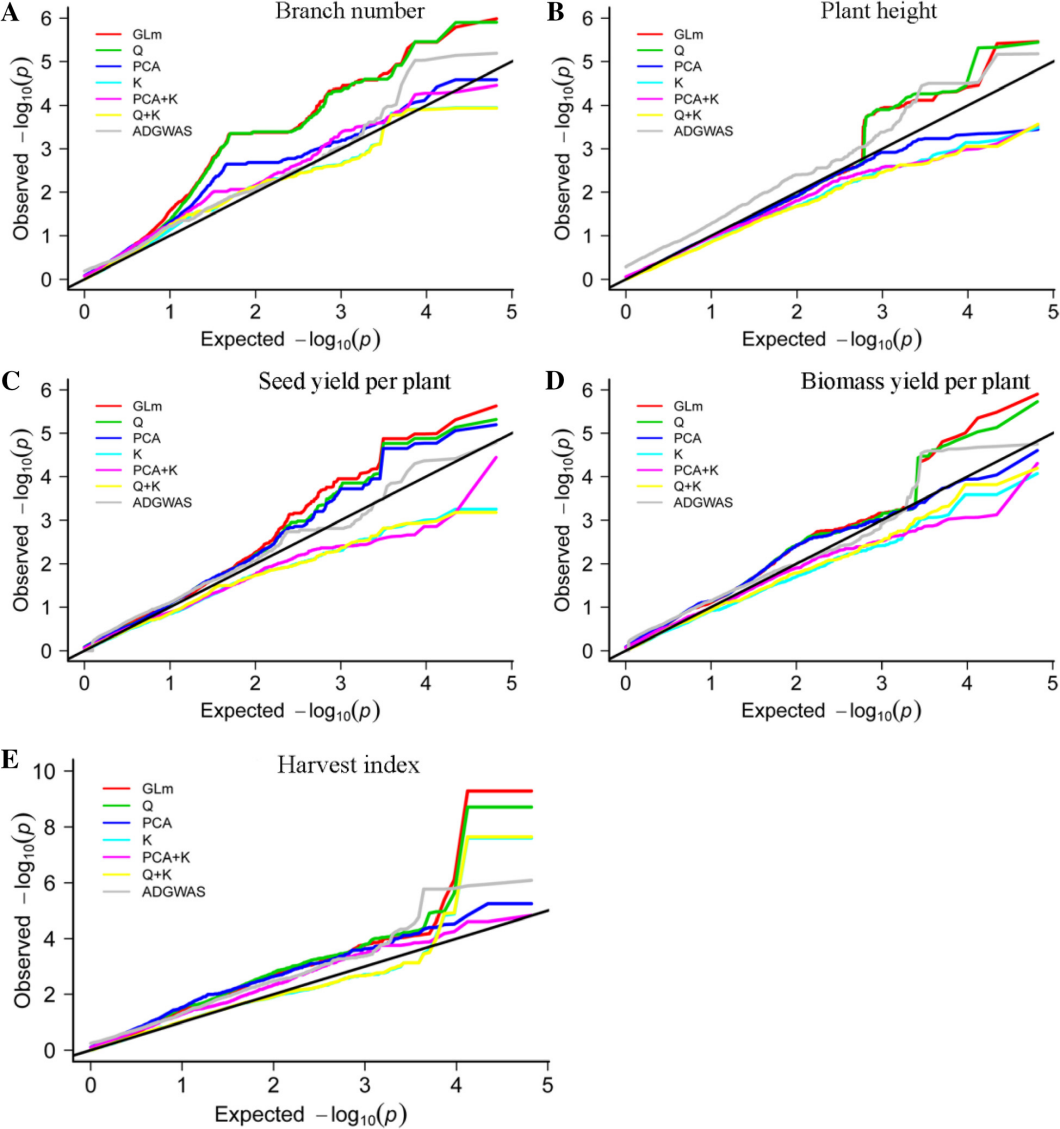
Quantile–quantile plots of estimated−log10 (P) from association analysis of harvest index (HI) and associated traits. (**A**) Branch number. (**B**) Plant height. (**C**) Seed yield per plant. (**D**) Biomass yield per plant. (**E**) Harvest index. Black line represents expected P values with no associations. Red line represents observed P values using GLM model. Green line represents observed P values using Q model. Blue line represents observed P values using PCA model. Cyan line represents observed P values using K model. Pink line represents observed P values using the PCA+K model. Orange line represents observed P values using Q+K model. Gray line represents observed P values using ADGWAS model. (Color figure online)

Association analyses were performed for the five traits to evaluate the effects of population structure (Q, PC) and familial relationship (K) on controlling false associations. Among all the tested models (GLM, Q, PCA, K, PCA+K, Q+K and ADGWAS), the P values from the ADGWAS model showed the best fit to the expected P values for all five traits (Fig. 2A, B, C, D, E). Thus, the ADGWAS model was selected to conduct association mapping for HI and its related traits.

### Association mapping for complex traits

**Fig. 3:**
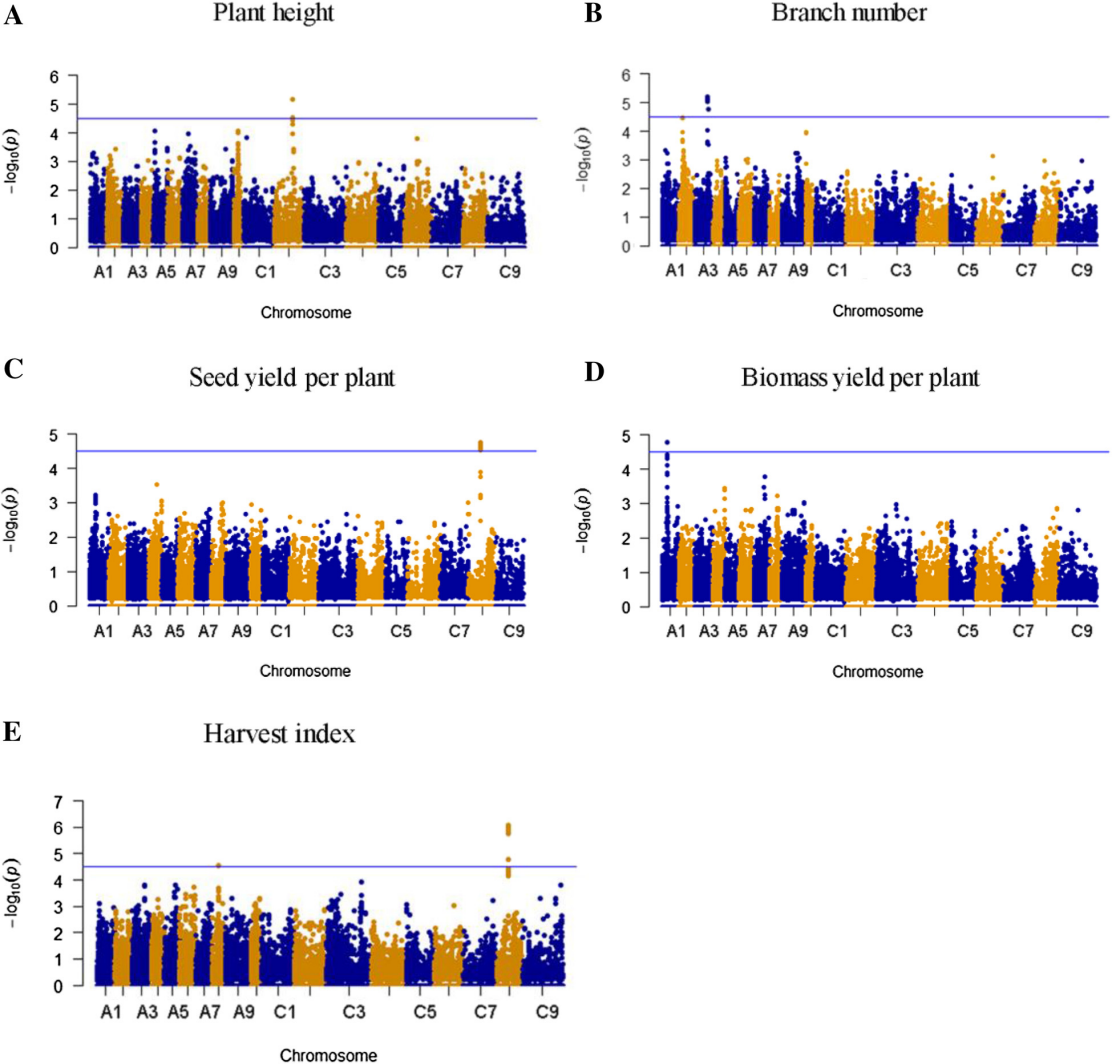
Manhattan and quantile–quantile plots generated from genome-wide association analysis results for complex traits. (**A**) Plant height. (**B**) Branch number. (**C**) Seed yield per plant. (**D**) Biomass yield per plant. (**E**) Harvest index. Blue horizontal line depicts Bonferroni significance threshold (2.79 E-5)

In total, 35,791 polymorphisms with minor allele frequency (MAF) ≥ 0.05 were selected for association mapping. Of these, 29 SNPs were identified to be highly significantly associated with the five complex traits (P < 2.79 E-05; Table 2; Fig. 3).

**Table 2:** Summary of SNPs significantly associated with complex traits

Trait	SNP	Chromosome	Position	Allele	MAF	*P* Value	R^2^ (%)
PH	Bn-scaff_16300_1-p216539	C2	31484854	[T/C]	0.22	6.63E-06	1.19
	Bn-scaff_16300_1-p222857	C2	31483855	[T/G]	0.23	6.82E-06	
BN	Bn-A03-p22138059	A3	22138109	[T/C]	0.24	6.45E-06	2.04
	Bn-A03-p22145945	A3	22145995	[T/C]	0.24	7.23E-06	
	Bn-A03-p22237846	A3	22237796	[A/G]	0.23	8.40E-06	
	Bn-A03-p22238801	A3	22238851	[A/G]	0.25	9.36E-06	
	Bn-A03-p22149000	A3	22148950	[T/G]	0.25	9.40E-06	
	Bn-A03-p14121492	A3	14121442	[T/C]	0.3	1.74E-05	
SY	Bn-scaff_16962_1-p506943	C8	18144556	[A/G]	0.34	1.78E-05	3.96
	Bn-scaff_16962_1-p519147	C8	18381806	[T/C]	0.34	2.01E-05	
	Bn-scaff_16962_1-p494346	C8	18403153	[A/G]	0.33	2.10E-05	
	Bn-scaff_16962_1-p378035	C8	18497622	[T/G]	0.35	2.24E-05	
	Bn-scaff_16962_1-p504805	C8	18395390	[A/G]	0.34	2.31E-05	
	Bn-scaff_16962_1-p543498	C8	18359355	[A/G]	0.33	2.31E-05	
	Bn-scaff_16962_1-p359041	C8	18499598	[T/C]	0.35	2.55E-05	
	Bn-scaff_16962_1-p376380	C8	18498612	[T/G]	0.36	2.55E-05	
	Bn-scaff_16962_1-p432767	C8	18466493	[A/G]	0.36	2.55E-05	
	Bn-scaff_16962_1-p441134	C8	18458145	[T/G]	0.36	2.55E-05	
	Bn-scaff_16962_1-p542817	C8	18365405	[A/G]	0.34	2.66E-05	
BY	Bn-A01-p8418102	A1	8418052	[A/G]	0.39	1.63E-05	0.76
HI	Bn-scaff_16231_1-p2306931	C8	18546937	[T/C]	0.49	8.14E-07	3.42
	Bn-scaff_16962_1-p426711	C8	18472658	[T/C]	0.35	1.12E-06	
	Bn-scaff_16231_1-p2303901	C8	18301213	[A/G]	0.5	1.28E-06	
	Bn-scaff_16962_1-p378035	C8	18497622	[T/G]	0.35	1.59E-06	
	Bn-scaff_16962_1-p359041	C8	18499598	[T/C]	0.35	1.68E-06	
	Bn-scaff_16962_1-p376380	C8	18498612	[T/G]	0.36	1.68E-06	
	Bn-scaff_16962_1-p432767	C8	18466493	[A/G]	0.36	1.68E-06	
	Bn-scaff_16962_1-p441134	C8	18458145	[T/G]	0.36	1.68E-06	
	Bn-scaff_16962_1-p351914	C8	18506723	[T/G]	0.48	1.71E-05	

*MAF* Minor allele frequency, *R* ^*2*^ *(%)* Amount of phenotypic variation for each trait explained by multiple SNPs.

For PH, two SNPs (Bn-scaff_16300_1-p216539 and Bn-scaff_16300_1-p222857) were detected on C2, and these SNPs explained 1.19 % of the total phenotypic variance. For BN, six significant SNPs were detected on A3, and could explain 2.04 % of the total phenotypic variance. For BY, only one SNP (Bn-A01-p8418102) on A1 was detected (P = 1.63 E-05). This SNP could only explain 0.76 % of the total phenotypic variance. For SY, 11 SNPs on C8 were detected. These SNPs could explain 3.96 % of the total phenotypic variance. For HI, nine SNPs on C8 were detected. These SNPs could explain 3.42 % of the total phenotypic variance. Among these SNPs, five were co-associated with HI and SY, but there were no SNPs that were co-associated with HI and the other three traits (PH, BN and BY). LD analyses showed that r^2^ values of most pairs of the multiple SNPs on C8 were >0.20, and those of most pairs of multiple SNPs on A3 were >0.50, except the Bn-A03-p14121492 (Additional file 4: Figure S1). These results suggested that the majority of the multiple SNPs on C8 and A3 were in high linkage disequilibrium with each other [38].

## Discussion

### Phenotypic variations in harvest index

For crops such as rice, wheat, barley and maize, HI has been shown to be a variable factor, with a value of approximately 0.50 [39]. Soybean, one of the most important oil crops, has a HI ranging from 0.4 to 0.6 [40, 41], and the HI has been successfully maximized during breeding [42]. However, the average HI of *B. napus* was reported to be approximately 0.2–0.3 [43, 44]. In our study, the range of the HI of the GWAS population could be >0.30 in a single environment (Table 1), indicating that there is still great potential for HI improvement. Therefore, increasing HI might be an alternative strategy to increase seed yield gain in *B. napus*.

Quantitative traits related to harvest index show a range of sensitivities to environmental factors. Yang *et al.* [39] showed that proper crop management holds great promise to enhance the HI of rice. D’Andrea [45] evaluated the effects of the genotype and environment interaction on variations in plant grain yield, HI and biomass production at maturity in maize. In the present study, HI and its associated traits were significantly affected by the environment and genotype (Additional file 2: Table S2), consistent with the results of a study on rice [46].

The correlations among HI and other traits indicated that HI was negatively or not strongly positively correlated with PH (Additional file 3: Table S3). Interestingly, the HI of rice has increased primarily due to the introduction of the semi-dwarf gene [47, 48]. In this study, HI was strongly significantly correlated with SY in multiple environments, but exhibited a complex relationship with BY and BN (Additional file 3: Table S3). Rapeseed plants normally have flourishing leaves, pods, flowers and other above-ground green tissues, but their seeds are often only partially filled, probably because most of the photosynthetic products are stored in flowers and pods, rather than seeds. Also, the photosynthetic products in pods are not efficiently transported to seeds, resulting in poor seed filling. These results have laid the genetic basis for increasing HI in rapeseed by properly decreasing PH and/or improving transport efficiency from pods to seeds.

### Population structure and model comparison

Our results demonstrate the significant potential effect of population structure on the false positive rate in association mapping. Among the seven models, the GLM model performed similarly to the Q model, K model and Q+K model for all five traits (Fig. 2A, B, C, D, E). The PCA model, PCA+K model, K model and Q+K model performed better than the GLM model and the Q model, and might indicate potentially false negatives for BN, PH and BY (Fig. 2A, B, D). For HI, the K and Q+K models did not perform better than the GLM and Q models, and PCA, and PCA+K models could reduce false positives, but may have indicated potentially false negatives (Fig. 2E). Compared with the other six models, ADGWAS showed the best fit for the association analysis (Fig. 2). Therefore, the efficiency of the seven models varied from trait to trait. To reduce the frequencies of false positives and false negatives, the ADGWAS model was used for the association analysis of HI and its related traits.

Several methods have been proposed to deal with false positives caused by population structure. Flowering time is likely involved in local adaptation, and removing the genetically distinct and phenotypically extreme accessions can indeed reduce the false positive rate [26]. However, no information is available about other traits such as yield and resistance. Haplotype-based *de novo* assembly of the sequencing data is an alternative approach to estimate the effect of population structure on association statistics [24]. We did not try this approach for two reasons: first, rapeseed is an allopolyploid species with a complex genome structure and a number of repeat sequences. Second, to date, there have been no reports on map-based cloning of a causal gene in a QTL of rapeseed. The combination of association mapping and linkage mapping can provide both the power and resolution needed to detect QTL of interest, and have proven to be more successful than either strategy alone [28]. Thus, the linkage mapping strategy will be used in our future work to identify the potential candidate genes by map-based cloning.

### Genetic dissection of harvest index

The HI is an integrative trait including the net effects of all physiological processes during the crop cycle, and is correlated with yield-related traits. The phenotypic expression of HI is theoretically affected by genes responsible for yield-related traits. Li *et al.* [21] detected an associated SNP Bn-A10-p12639538 at 2.67 Mb of A7, which explained 4.9 % of the total seed weight variation in rapeseed. Cai *et al.* [22] identified 43 loci (*P* < 0.001) associated with plant height, first branch height, inflorescence length, silique length, seeds per silique and seed weight in rapeseed. Li *et al.* [23] identified 13 consensus QTL for seed weight and 9 QTL for silique length; these QTL explained 0.7–67.1 % and 2.1–54.4 % of the phenotypic variance in seed weight and silique length in rapeseed, respectively. In our previous study, a functional marker derived from the sucrose transporter gene (*SUT*) was co-localized with a seed yield QTL in *B. napus* [49], and allelic variations in *BnA7.SUT1* were associated with seed yield-related traits (*BnA7.SUT1.b* and its promoter were linked to higher seed yield, while *BnA7.SUT1.a* was associated with increased seed weight) [50]. However, no QTL or loci was identified to be directly or indirectly associated with HI in rapeseed, and no QTL, or loci was common with the SNPs detected in the present study. To our knowledge, this is the first report of a QTL analysis of HI-related traits in rapeseed*.* In the present study, nine SNPs were detected to significantly associate with HI, and could explain 3.42 % of the observed variation (Table 2). These results have confirmed that HI is a complex polygenic phenomenon in rapeseed, like in rice [51]. Five SNPs were detected to significantly associate with both HI and SY. These SNPs might represent a shared genetic mechanism between the HI and SY in rapeseed. Additionally, PH was correlated with HI, but no SNP was simultaneously associated with PH and HI, possibly because the SNPs associated with PH did not directly affect HI. None of the nine HI SNPs co-located with BY and BN SNPs, which was largely consistent with the observation that there was a significant phenotypic correlation between these traits only in E3, and not in the other three environments (Additional file 3: Table S3).

## Conclusions

A whole genome scan identified a total of nine significant SNPs for HI. The results can contribute to a better understanding of natural variations of HI, and provide a useful resource for marker-assisted selection breeding.

## Availability of supporting data

The data sets supporting the results of this article are included within the article and its additional files.

## Additional files

Additional file 1: Table S1. Phenotype data for five traits used in association analysis. Additional file 2: Table S2. ANOVA for the five traits. Additional file 3: Table S3. Correlation analysis between HI and other traits. Additional file 4: Figure S1. Linkage disequilibrium (LD) analysis around multiple SNPs on C8, A3 found associations with traits (SY, HI and BN).
