# Supplementary material for: Unravelling the complex trait of harvest index in rapeseed (Brassica napus L.) with association mapping
Source: BMC Genomics. 2015 May 12;16(1):379. doi: 10.1186/s12864-015-1607-0 (PMC4427920; doi:10.1186/s12864-015-1607-0)
Supplement: Additional file 2: Table S2. — ANOVA for the five traits. [file 12864_2015_1607_MOESM2_ESM.doc]

**Table S2.** ANOVA for the five traits.

| Trait | Source | F value | Pr>F |
| --- | --- | --- | --- |
| PH | G | 3.31 | 0.000 |
|  | E | 9.73 | 0.002 |
| BN | G | 1.41 | 0.000 |
|  | E | 258.99 | 0.000 |
| BY | G | 1.32 | 0.017 |
|  | E | 87.39 | 0.000 |
| SY | G | 1.25 | 0.042 |
|  | E | 12.75 | 0.000 |
| HI | G | 1.27 | 0.032 |
|  | E | 121.51 | 0.000 |

Source: G, genotype; E, environment
