# Supplementary material for: Unravelling the complex trait of harvest index in rapeseed (Brassica napus L.) with association mapping
Source: BMC Genomics. 2015 May 12;16(1):379. doi: 10.1186/s12864-015-1607-0 (PMC4427920; doi:10.1186/s12864-015-1607-0)
Supplement: Additional file 3: Table S3. — Correlation analysis between HI and other traits. [file 12864_2015_1607_MOESM3_ESM.doc]

**Table S3.** Correlation analysis between HI and other traits.

| Trait | Env. | PH | BN | BY | SY |
| --- | --- | --- | --- | --- | --- |
| HI | E1 | ＊ | 0.10 | -0.10 | 0.34** |
|  | E2 | ＊ | 0.03 | 0.19* | 0.63** |
|  | E3 | -0.29** | 0.23** | 0.37** | 0.83** |
|  | E4 | 0.01 | 0.05 | 0.12 | 0.43** |

* Significant at P=0.05

**Significant at P=0.01

＊: data not collected.
