# Supplementary material for: Unravelling the complex trait of harvest index in rapeseed (Brassica napus L.) with association mapping
Source: BMC Genomics. 2015 May 12;16(1):379. doi: 10.1186/s12864-015-1607-0 (PMC4427920; doi:10.1186/s12864-015-1607-0)
Supplement: Additional file 4: Figure S1. — Linkage disequilibrium (LD) analysis around multiple SNPs on C8, A3 found associations with traits (SY, HI and BN). [file 12864_2015_1607_MOESM4_ESM.doc]

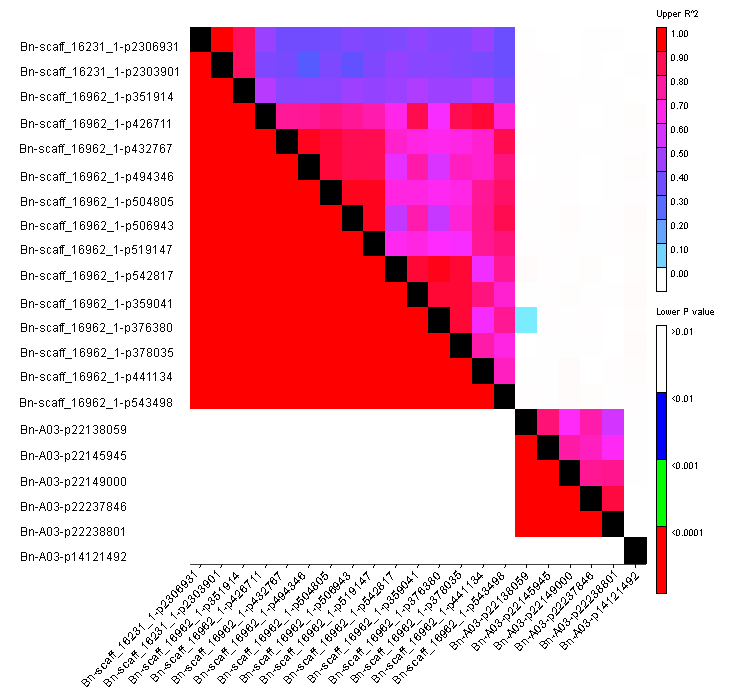


**Figure S1. Linkage disequilibrium (LD) analysis around multiple SNPs on C8, A3 found associations with traits (SY, HI, and BN).** Cells above diagonal show squared allele frequency correlation (r^2^); cells below diagonal represent significance level of LD (Fisher’s exact test).
